# Além da Curva de Aprendizado: Integrando a Litotripsia Intravascular Coronária às Estratégias Contemporâneas de Modificação do Cálcio

**DOI:** 10.36660/abc.20260619

**Published:** 2026-09-21

**Authors:** Ricardo Czarnobai Soccol, Paulo R. A. Caramori

**Affiliations:** 1 Hospital São Lucas da PUCRS Porto Alegre RS Brasil Hospital São Lucas da PUCRS, Porto Alegre, RS – Brasil

**Keywords:** Doença da Artéria Coronariana, Angioplastia, Litotripsia

Poucas tecnologias transformaram tão profundamente o tratamento das lesões coronárias intensamente calcificadas quanto a litotripsia intravascular coronária (IVL). Utilizando ondas de pressão acústica localizadas, emitidas por meio de um balão semi-complacente de baixa pressão, a IVL fratura depósitos de cálcio superficiais e profundos, melhorando a complacência do vaso e minimizando a lesão vascular. Diferentemente da aterectomia, a IVL modifica a placa calcificada sem a necessidade de remoção de tecido ou dispositivos rotacionais de alta velocidade, oferecendo uma abordagem baseada em balão que simplifica o preparo da lesão e facilita a expansão ideal do stent. A segurança e a eficácia da IVL foram estabelecidas pelo programa clínico DISRUPT CAD, que demonstrou alta taxa de sucesso do procedimento, fratura consistente do cálcio e resultados clínicos favoráveis em curto prazo em um amplo espectro de lesões severamente calcificadas.^1,2^ Em última análise, a combinação de uma plataforma de balão familiar ao cardiologista intervencionista com evidências clínicas consistentes provenientes do programa DISRUPT CAD abriu caminho para a rápida incorporação da IVL à intervenção coronária percutânea (ICP) contemporânea.

A eficácia clínica, no entanto, é apenas o começo. O verdadeiro teste de qualquer tecnologia disruptiva reside na sua incorporação à prática cotidiana. Como os operadores a incorporam em procedimentos cada vez mais complexos? Como a seleção de pacientes evolui com o aumento da experiência? E onde a tecnologia se encaixa, em última análise, em um arsenal crescente de técnicas de modificação do cálcio? Essas questões práticas são frequentemente mais relevantes para a prática clínica diária do que aquelas abordadas nos estudos clínicos pivotais e foram recentemente abordadas no documento de consenso clínico da EAPCI sobre o manejo de estenoses coronárias intensamente calcificadas.^3^

Nesta edição do Arquivos Brasileiros de Cardiologia, Prado et al. apresentam o primeiro registro brasileiro abrangente que avalia sistematicamente a implementação da IVL coronária na prática clínica de rotina em um centro de alto volume com vasta experiência em ICP complexa.^4^ Mais do que simplesmente relatar os resultados dos procedimentos, os autores descrevem como a utilização da IVL evoluiu após sua introdução na prática clínica. É importante ressaltar que o registro reflete a complexidade da ICP contemporânea, incluindo pacientes com síndromes coronárias agudas, doença do tronco da coronária esquerda, doença renal crônica avançada, reestenose intra-stent, lesões de bifurcação e estratégias combinadas de modificação da placa — cenários clínicos que permanecem sub-representados nos principais ensaios clínicos de IVL.^2^

À primeira vista, a principal mensagem deste registro parece ser a demonstração de uma curva de aprendizado favorável. No entanto, acreditamos que sua maior contribuição reside em outro aspecto. A lição mais importante não é que os operadores se tornaram tecnicamente mais proficientes, mas sim que sua tomada de decisão clínica se tornou progressivamente mais refinada. A IVL é tecnicamente simples para operadores de ICP experientes. O que evolui com a experiência é o julgamento, e não a técnica: identificar as lesões com maior probabilidade de se beneficiarem da IVL, reconhecer quando dispositivos alternativos de modificação da placa devem ser empregados, integrar a imagem intracoronária ao planejamento do procedimento^5^ e determinar quando tecnologias complementares devem ser combinadas em vez de serem vistas como opções concorrentes.^3^ A redução das complicações angiográficas observada ao longo do tempo provavelmente reflete mais essa evolução do raciocínio clínico do que o domínio técnico da tecnologia em si.

Essa observação reflete uma transformação mais ampla no manejo da calcificação coronária. A ICP contemporânea não é mais definida pela seleção do “melhor” dispositivo de modificação do cálcio, mas sim pela escolha da estratégia mais apropriada.^3,6^ A morfologia do cálcio, a arquitetura vascular, os objetivos do procedimento e a imagem intracoronária ditam cada vez mais quais tecnologias devem ser utilizadas^5^e, talvez ainda mais importante, em que combinação. Nesse contexto, a IVL não deve ser vista como concorrente da aterectomia ou dos balões especiais, mas como parte integrante de um arsenal complementar de estratégias para modificação do cálcio. Abordagens híbridas que combinam IVL com aterectomia ou balões especiais exemplificam essa filosofia, reconhecendo que diferentes tecnologias abordam aspectos mecânicos e biológicos distintos da placa calcificada, em vez de competirem pela mesma indicação.^3^

O registro também ilustra outra característica marcante da adoção bem-sucedida de tecnologia. À medida que a experiência institucional se acumulava, a seleção de pacientes se tornou cada vez mais refinada, apesar da crescente complexidade anatômica. Essa observação sugere que a maturidade clínica não se caracteriza pela expansão indiscriminada das indicações, mas sim por uma identificação mais precisa dos cenários clínicos nos quais a IVL proporciona um benefício incremental significativo. Tal refinamento representa um estágio esperado — e desejável — na evolução de qualquer nova tecnologia.

Como acontece com qualquer tecnologia em rápida evolução, questões importantes permanecem. Estudos futuros devem se concentrar menos em estabelecer que a IVL funciona^2,7,8^— o que já foi demonstrado de forma convincente — e mais em definir quando ela deve ser usada, em quem e em combinação com quais técnicas complementares.^3,9^ Estudos comparativos de estratégias de modificação do cálcio, algoritmos de tratamento guiados por imagem e resultados a longo prazo refinarão ainda mais o papel da IVL na ICP contemporânea.^10^À medida que novas plataformas se tornam disponíveis, a ênfase naturalmente mudará da experiência inicial em centros especializados para uma implementação mais ampla em diversos sistemas de saúde. O próximo desafio será determinar se os resultados favoráveis alcançados em instituições especializadas podem ser reproduzidos de forma consistente na prática clínica de rotina.

Embora este registro brasileiro documente uma curva de aprendizado favorável, sua maior contribuição reside em registrar a transição da litotripsia intravascular da inovação para a integração à prática clínica. À medida que nossa compreensão do cálcio coronário continua a evoluir, o futuro do preparo de lesões provavelmente dependerá menos de dispositivos individuais e mais de combinações criteriosas e guiadas por imagem de tecnologias complementares, adaptadas às características específicas de cada lesão. A curva de aprendizado da IVL pode se mostrar surpreendentemente curta. O domínio de sua utilização ideal, entretanto, está apenas começando.
