# Supplementary material for: Além da Curva de Aprendizado: Integrando a Litotripsia Intravascular Coronária às Estratégias Contemporâneas de Modificação do Cálcio
Source: Arq Bras Cardiol. 2026 Sep 21;123(8):e20260619. [Article in Portuguese] doi: 10.36660/abc.20260619 (PMC13649073; doi:10.36660/abc.20260619)
Supplement: Translation

# Translation: Beyond the Learning Curve: Integrating Coronary Intravascular Lithotripsy into Contemporary Calcium Modification

**DOI:** 10.36660/abc.20260619i

**Authors:** Ricardo Czarnobai Soccol, Paulo R. A. Caramori

**Affiliations:** 1 Brazil Hospital São Lucas da PUCRS, Porto Alegre, RS – Brazil

**Keywords:** Coronary Artery Disease, Angioplasty, Lithotripsy

Few technologies have transformed the treatment of heavily calcified coronary lesions as profoundly as coronary intravascular lithotripsy (IVL). Using localized acoustic pressure waves delivered through a low-pressure, semi-compliant balloon, IVL fractures both superficial and deep calcium deposits, improving vessel compliance while minimizing vascular injury. Unlike atherectomy, IVL modifies calcified plaque without tissue debulking or high-speed rotational devices, offering a balloon-based approach that simplifies lesion preparation while facilitating optimal stent expansion. The safety and effectiveness of IVL were established by the DISRUPT CAD clinical program, which demonstrated high procedural success, consistent calcium fracture, and favorable short-term clinical outcomes across a broad spectrum of severely calcified lesions.^1,2^ Ultimately, it was the combination of a familiar balloon-based platform and the consistent clinical evidence from the DISRUPT CAD program that paved the way for the rapid adoption of IVL in contemporary percutaneous coronary intervention (PCI).

Clinical efficacy, however, is only the beginning. The true test of any disruptive technology lies in its translation into everyday practice. How do operators incorporate it into increasingly complex procedures? How does patient selection evolve with growing experience? And where does the technology ultimately fit within an expanding armamentarium of calcium modification techniques? These practical questions are often more relevant to daily practice than those addressed by pivotal clinical trials and have recently been addressed in the EAPCI clinical consensus statement on the management of heavily calcified coronary stenoses.^3^

In this issue of the *Arquivos Brasileiros de Cardiologia*, Prado et al. present the first comprehensive Brazilian registry systematically evaluating the implementation of coronary IVL in routine clinical practice at a high-volume center with extensive expertise in complex PCI.^4^ Rather than simply reporting procedural outcomes, the investigators describe how IVL adoption evolved after its introduction into clinical practice. Importantly, the registry reflects the complexity of contemporary PCI, including patients with acute coronary syndromes, left main disease, advanced chronic kidney disease, in-stent restenosis, bifurcation lesions, and combined plaque-modification strategies — clinical scenarios that remain underrepresented in the pivotal IVL trials.^2^

At first glance, the main message of this registry appears to be the demonstration of a favorable learning curve. However, we believe its greatest contribution lies elsewhere. The most important lesson is not that operators became technically more proficient, but that their clinical decision-making became progressively more refined. IVL is technically straightforward for experienced PCI operators. What evolves with experience is judgment rather than technique: identifying the lesions most likely to benefit from IVL, recognizing when alternative plaque-modification devices should be employed, integrating intracoronary imaging into procedural planning,^5^ and determining when complementary technologies should be combined rather than viewed as competing options.^3^ The reduction in angiographic complications observed over time likely reflects this evolution in clinical thinking more than technical mastery itself.

This observation mirrors a broader transformation in the management of coronary calcification. Contemporary PCI is no longer defined by selecting the “best” calcium-modification device, but by choosing the most appropriate strategy.^3,6^ Calcium morphology, vessel architecture, procedural objectives, and intracoronary imaging increasingly dictate which technologies should be used^5^and, perhaps more importantly, in what combination. In this context, IVL should not be viewed as a competitor to atherectomy or specialty balloons, but as an integral component of a complementary calcium-modification toolbox. Hybrid approaches combining IVL with atherectomy or specialty balloons exemplify this philosophy, recognizing that different technologies address distinct mechanical and biological aspects of calcified plaque rather than competing for the same indication.^3^

The registry also illustrates another hallmark of successful technology adoption. As institutional experience accumulated, patient selection became increasingly refined despite growing anatomical complexity. This observation suggests that clinical maturity is characterized not by indiscriminately expanding indications, but through a more precise identification of the clinical scenarios in which IVL provides meaningful incremental benefit. Such refinement represents an expected—and desirable—stage in the evolution of any novel technology.

As with any rapidly evolving technology, important questions remain. Future studies should focus less on establishing that IVL works^2,7,8^— which has already been convincingly demonstrated — and more on defining when it should be used, in whom, and in combination with which complementary techniques.^3,9^Comparative studies of calcium-modification strategies, imaging-guided treatment algorithms, and longer-term outcomes will further refine the role of IVL in contemporary PCI.^10^ As additional platforms become available, the emphasis will naturally shift from early experience in expert centers to broader implementation across diverse healthcare systems. The next challenge will be determining whether the favorable outcomes achieved in specialized institutions can be consistently reproduced in routine clinical practice.

Although this Brazilian registry documents a favorable learning curve, its greatest contribution is capturing the transition of IVL from innovation to integration. As our understanding of coronary calcium continues to evolve, the future of lesion preparation will likely depend less on individual devices and more on thoughtful, imaging-guided combinations of complementary technologies tailored to specific lesion characteristics. The learning curve of IVL may prove remarkably short. Mastering its optimal use has only just begun.
